# Electrically Guided DNA Immobilization and Multiplexed DNA Detection with Nanoporous Gold Electrodes

**DOI:** 10.3390/nano8050351

**Published:** 2018-05-21

**Authors:** Jovana Veselinovic, Zidong Li, Pallavi Daggumati, Erkin Seker

**Affiliations:** 1 Department of Chemical Engineering, University of California—Davis, Davis, CA 95616, USA; jveselinovic@ucdavis.edu; 2 Department of Biomedical Engineering, University of California—Davis, Davis, CA 95616, USA; zdli@ucdavis.edu; 3 Department of Electrical and Computer Engineering, University of California—Davis, Davis, CA 95616, USA; pdaggumati@ucdavis.edu

**Keywords:** nanoporous gold, electrophoresis, electrochemical biosensor, multiplexed detection, nucleic acids

## Abstract

Molecular diagnostics have significantly advanced the early detection of diseases, where the electrochemical sensing of biomarkers (e.g., DNA, RNA, proteins) using multiple electrode arrays (MEAs) has shown considerable promise. Nanostructuring the electrode surface results in higher surface coverage of capture probes and more favorable orientation, as well as transport phenomena unique to nanoscale, ultimately leading to enhanced sensor performance. The central goal of this study is to investigate the influence of electrode nanostructure on electrically-guided immobilization of DNA probes for nucleic acid detection in a multiplexed format. To that end, we used nanoporous gold (np-Au) electrodes that reduced the limit of detection (LOD) for DNA targets by two orders of magnitude compared to their planar counterparts, where the LOD was further improved by an additional order of magnitude after reducing the electrode diameter. The reduced electrode diameter also made it possible to create a np-Au MEA encapsulated in a microfluidic channel. The electro-grafting reduced the necessary incubation time to immobilize DNA probes into the porous electrodes down to 10 min (25-fold reduction compared to passive immobilization) and allowed for grafting a different DNA probe sequence onto each electrode in the array. The resulting platform was successfully used for the multiplexed detection of three different biomarker genes relevant to breast cancer diagnosis.

## 1. Introduction

**Figure A1:**
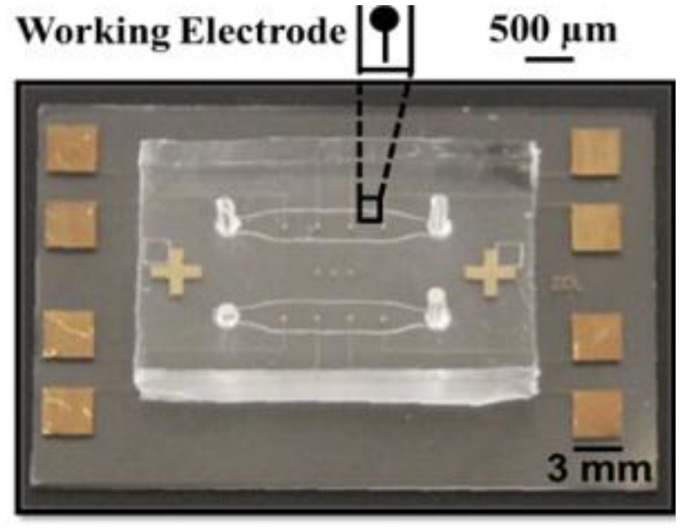
Optical image of a np-Au multielectrode array integrated with polydimethylsiloxane (PDMS) microfluidic channels that house the micro-scale electrodes.

Nucleic acid-based biosensors offer high selectivity and sensitivity for biomolecule detection and, consequently, they have been instrumental in point-of-care diagnostic platforms engineered during the past decade [1]. DNA microarrays dramatically accelerated the development of technologies for multiplexed pathogen detection and cancer biomarker identification on a single chip [2]. Multiplexed detection of different biomarkers is crucial for a more conclusive diagnosis, for example in the case of breast cancer [3]. On-chip hybridization in DNA microarrays is usually monitored by fluorescence or chemiluminescence, and less commonly by electrochemical markers [4,5]. Electrochemical DNA sensors are of particular interest for their facile integration with electronic instrumentation, thereby being conducive to implementation as point-of-care platforms. Traditionally, electrochemical DNA sensors have employed planar gold (pl-Au) working electrodes, where steric hindrance in target molecules approaching the surface-bound capture probes has limited sensitivity, selectivity, and limit of detection (LOD) [6]. Nanostructuring of the working electrode with different coating types (carbon nanotubes [7], gold nanorods [8], palladium dendritic nanostructures [9]) provided significant improvements in sensor performance [10,11,12,13] through several mechanisms unique to nano-scale, including the display of capture probes with more favorable orientation [6,14]. We previously demonstrated that nanoporous gold (np-Au) as a working electrode, obtained by a microfabrication-compatible self-assembly process, offered higher hybridization efficiency compared to its planar counterpart, biofouling resilience due to size-selective permittivity to small redox molecules and fibrillar nucleic acid analytes, tunable dynamic ranges due to morphology-dictated transport of target nucleic acids, and sequence-specific purification of short nucleic acid fragments in complex biological samples [15,16,17,18]. Microfluidic encapsulation and miniaturization of the electrodes, as well as entrapment of DNA probes in polymer coatings [19], provides an additional layer of sensor enhancement by requiring less sample volume, better control on reagent delivery, and faster sensor response [2,20]. A significant challenge for microfluidics-based sensor platforms is the difficulty in immobilizing different DNA probes on individual electrodes, as the electrodes are too small to manually pipette probe DNA solutions without cross-contamination with the other electrodes. Electro-grafting has been shown to be a versatile solution, as the negatively-charged backbone of the DNA probes can be leveraged to direct probe immobilization to a specific electrode by holding it at a positive potential with respect to the other electrodes in the array [21,22]. However, there is a lack of fundamental understanding of the factors that affect electrically-guided grafting of probe DNA on multiple electrode arrays (MEAs) consisting of nanostructured electrodes, where DNA immobilization takes significantly longer due to hindered transport of molecules within the nanoscale geometries [23,24,25]. This study aims to provide insight into the effect of the np-Au electrode nanostructuring on the electro-grafting of DNA probes by comparing their relative grafting density and immobilization kinetics as a function of different working electrode morphologies. Furthermore, by leveraging the advantages of nanostructured electrodes, electrically-guided grafting of probe DNA, and microfluidics, we report a hybrid approach for integrating np-Au multielectrode arrays (MEAs) within microfluidic channels, which enhances DNA sensor performance and facilitates multiplexed detection of multiple DNA biomarkers. More specifically, we demonstrate a microfluidic platform with an embedded np-Au MEA for electrochemical DNA sensing (Figure A1), where each electrode is individually functionalized with a DNA capture probe against common breast cancer-related genes (i.e., BRCA1, BRCA2, p53 [26,27]) by electrophoretic guidance of the probes to specific electrodes.

## 2. Materials and Methods

### 2.1. Chemicals and Reagents

Glass coverslips (22 mm × 22 mm × 0.15 mm) from Electron Microscopy Sciences, Hatfield, PA, USA, were used as substrates to deposit metal film electrodes embedded in both macro-scale (large volume) and micro-scale (microfluidic) electrochemical cells. Glass coverslips were cleaned with a piranha solution, composed of 1:4 volumetric ratio of hydrogen peroxide (30%) to sulfuric acid (96%), for 10 min, rinsed with deionized (DI) water, and dried under nitrogen flow. Sulfuric acid (96%) and hydrogen peroxide (30%) were purchased from J. T. Baker (Waltham, MA, USA). **CAUTION**: sulfuric acid, hydrogen peroxide, and nitric acid are extremely corrosive and should be handled with care after proper training. The gold, silver, and chrome targets (99.95% pure), used for metal film deposition, were purchased from Kurt J. Lesker (Phillipsburg, NJ, USA). Methylene blue (MB) and potassium ferricyanide [K_3_Fe(CN)_6_]/potassium ferrocyanide [K_4_Fe(CN)_6_], were purchased from Sigma-Aldrich, St. Louis, MO, USA. Tris(2-chloroethyl)-phosphate (TCEP) and magnesium chloride (MgCl_2_) were obtained from Thermo Fisher Scientific (Waltham, MA USA). Phosphate-buffered saline (PBS) was purchased from Corning (Corning, NY, USA) and has a composition of 137 mM NaCl, 2.7 mM KCl, 10 mM Na_2_HPO_4_, and 1.8 mM KH_2_PO_4_ with a pH of 7.4. Phosphate buffer (PB) was prepared from 1 M sodium phosphate dibasic and 1 M sodium phosphate monobasic solutions prepared from powder sodium phosphate dibasic and sodium phosphate monobasic obtained from Sigma-Aldrich dissolved in nuclease free water purchased from Thermo Fisher Scientific (Waltham, MA, USA). The thiolated 26-mer probe DNA was used to test the effect of the electrode morphology on the density of electro-grafted DNA probes. This same probe DNA and its complementary target were used to study hybridization efficiency of the macro-scale and micro-scale electrodes. For the study involving multiplexed detection of the breast cancer biomarkers, thiolated 19-mer BRCA1, 17-mer BRCA2 and 17-mer p53 probe/target matches were used. The DNA sequences are provided in Appendix A—Preparation of DNA Sensor.

### 2.2. Electrode Fabrication

**Figure A2:**
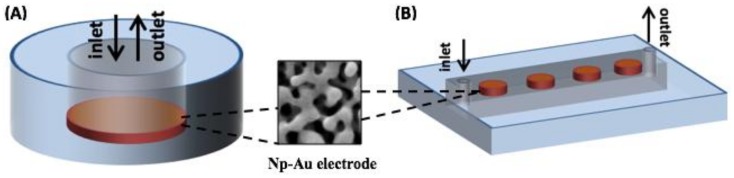
Schematic of (**A**) macro-scale electrochemical cell with np-Au electrode diameter of 4 mm and (**B**) microfluidic electrochemical cell with np-Au electrode diameter of 0.3 mm.

Nanoporous gold (np-Au) films for macro-scale electrochemical cell (Figure A2A) were prepared as described previously [16]. In summary, a 160 nm-thick chrome layer was sputtered on piranha-cleaned glass coverslips to promote adhesion between the glass substrate and the subsequent metallic layers. Following the sputtering of 80 nm-thick seed layer of gold, silver and gold were co-sputtered from different targets to obtain a 600 nm-thick alloy layer, with the alloy composition of 64% silver and 36% gold. All depositions were performed in argon at a pressure of 10 mTorr. The samples were then dealloyed in 70% nitric acid at 55 °C for 15 min to produce the np-Au films followed by rinsing with abundant DI water. To obtain samples with different pore morphologies (annealed np-Au), a group of dealloyed samples was thermally treated at 250 °C for 1.5 min on a hotplate. Planar gold electrodes were also fabricated by sputter-depositing a 50 nm-thick chrome adhesion layer followed by 250 nm-thick gold film onto piranha-cleaned glass coverslips.

**Figure 1:**
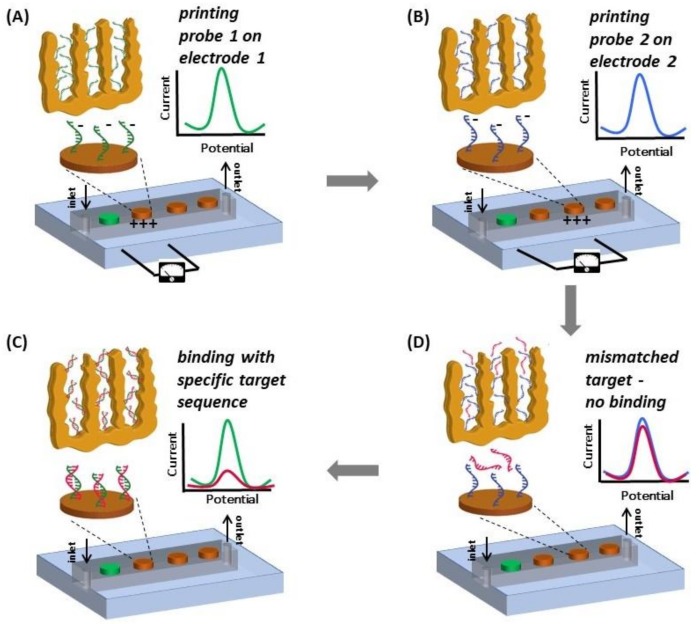
Scheme illustrating the concept of electrically-guided DNA grafting (note that the electrograms do not present actual data). (**A**) The microfluidic channel is filled with ssDNA probe 1 and a positive potential (0.8 V) is applied to electrode 1 to accelerate DNA transport and facilitate functionalization; (**B**) The channel is then filled with ssDNA probe 2 and probe 2 is grafted on electrode 2 in a similar fashion; (**C**) The device is then challenged with target DNA. Significant signal suppression is observed for the electrode with corresponding complementary probe; (**D**) No change in signal is seen on electrodes with mismatched probe-target pairs. All electrodes are functionalized and tested with both complementary and non-complementary target sequences.

**Figure A3:**
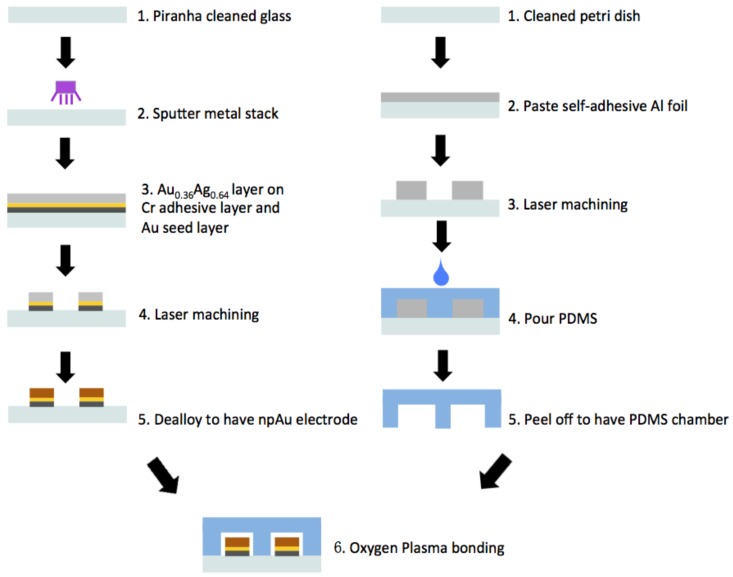
Process flow for the fabrication nanoporous gold (np-Au) multiple electrode arrays (MEAs) encapsulated with microfluidic channels. The MEAs were patterned by laser ablation of a blanket sputter-coated alloy films on glass slides. The molds for PDMS soft lithography were also fabricated by laser ablation. The np-Au MEAs were bonded to the PDMS device via oxygen plasma activation of the surfaces.

The np-Au MEAs (Figure A2B) were fabricated using a hybrid approach that merges rapid prototyping techniques [28] and corrosion-driven nanostructure self-assembly [29]. Briefly, glass microscope slides were piranha-cleaned followed by sputter-deposition of a stack of blanket metal layers (chrome adhesion layer, gold seed layer and gold-silver alloy layer) as described previously [16] and similar to how the samples for macro-scale electrochemical cell have been fabricated. The uniformly-coated microscope slides were patterned to create MEAs with electrode diameters of 300 µm using laser ablation for removing undesired regions of the blanket metal stack (Figure A3). The gold-silver MEAs were then dealloyed in heated nitric acid, where silver atoms dissolve in acid and gold atoms undergo surface diffusion to self-assemble a bi-continuous open-pore np-Au electrode [29]. For encapsulating the MEAs, polydimethylsiloxane (PDMS) microfluidic channels were fabricated via laser ablation and bonded to the MEAs via oxygen plasma activation of the surfaces [30] (see Appendix A—Fabrication of Microfluidic Channels for details). The integrated platform consisted of two microfluidic channels with four individually-addressable np-Au electrodes enclosed within each channel (Figure 1 and Figure A1). Planar gold counterparts were fabricated in a similar fashion. Ag/AgCl reference electrode and platinum wire counter electrodes were placed in the inlet and outlet ports of the microfluidic channel, respectively, and used for the electrochemical measurements.

### 2.3. Electrode Characterization

High-magnification images of the np-Au samples were captured via scanning electron microscope (SEM) (FEI Nova Nano-SEM430, Phenom World, Hillsboro, OR, USA) at 100 k× magnification to investigate np-Au electrode morphologies via top-view images and thickness via cross-sectional images. The average pore sizes for all samples were analyzed using ImageJ software as described previously (National Institutes of Health (NIH) shareware) [17]. The effective electrochemical surface area of different electrode morphologies in the macro-scale setup was obtained by performing cyclic voltammetry (CV) measurements in 0.5 M H_2_SO_4_ at a scan rate of 50 mV/s over the potential range of 0–1.8 V [31,32].

### 2.4. Electro-Grafting Protocol in Macro-Scale Electrochemical Cell

**Figure A4:**
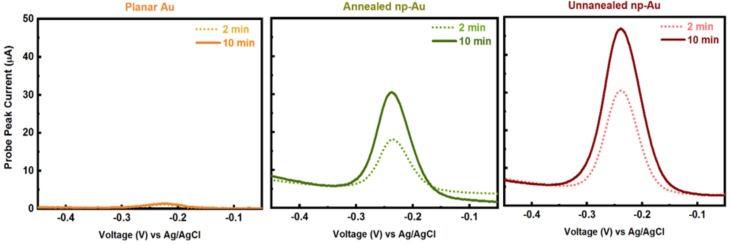
Example SWV raw data for two extreme electro-grafting times (2 and 10 min) used to generate Figure 3—Effect of electrode morphology (unannealed, annealed np-Au and pl-Au) and electro-grafting duration (1, 2, 5, 8, 10 min) on the peak current (surrogate for immobilized probe amount deducted from SWV measurements).

To test the effect of morphology on the density of electro-grafted DNA probes, a positive potential of 0.8 V (with respect to Ag/AgCl reference electrode of 3 M KCl) was applied to the working electrode in the macro-scale electrochemical cell (Figure A2B) via chronoamperometry for durations ranging between 1 and 10 min. The electrochemical cell was subsequently washed with phosphate buffer under open potential to remove nonspecifically-adsorbed ssDNA probes. The probe-modified electrodes were further treated with a backfill agent, 1 mM mercaptohexanol (MCH) prepared in PB for 20 min to obtain a well-ordered DNA−MCH monolayer. MCH incubation time for pl-Au electrodes was determined to be 10 min, as longer MCH incubation times resulted in desorption of DNA probes (see Appendix B—MCH Incubation Time Optimization). The electrodes were thoroughly rinsed with PB to remove non-specifically-bound DNA and MCH. Square wave voltammetry (SWV) using methylene blue (MB) redox marker was used for the characterization of electro-grafted probe DNAs (Figure A4) to minimize the non-faradaic current that typically dominates electrodes with high effective surface area (hence large capacitive current [33]). We specifically used MB redox marker for electrochemical DNA detection and quantification, as it has the ability to permeate the porous structure of the np-Au electrode before being fully depleted at the top surface due to its reaction-limited nature [16].

### 2.5. Hybridization Protocol for Microfluidic Electrochemical Cell

**Figure A6:**
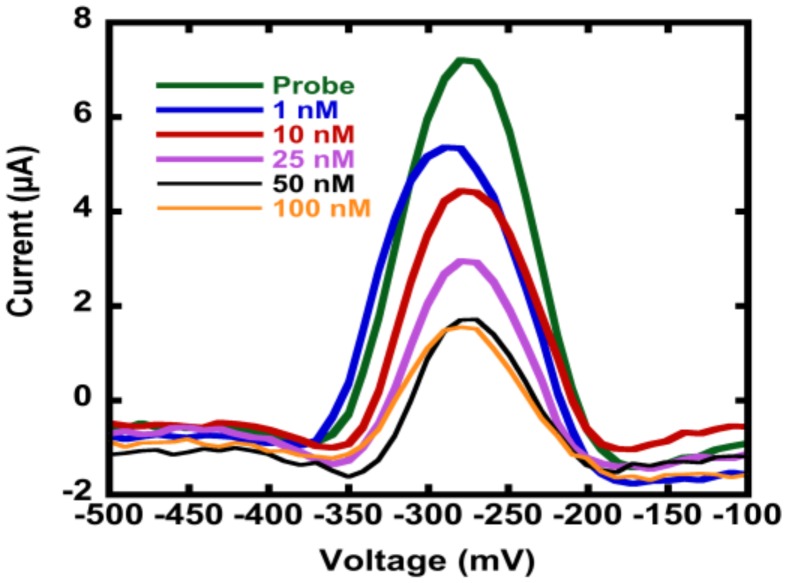
Square-wave voltammograms of probe DNA (26-mer) and probe/target DNA hybrid to show the detection limit of the probe functionalized np-Au MEA sensor upon exposure to different target concentrations. Decrease in the SWV peak indicates successful hybridization.

The np-Au electrodes (in both macro-scale electrochemical cell and microfluidic-encapsulated MEAs) were functionalized with ssDNA probes by passive incubation (Appendix A—Preparation of DNA Sensor) without applying an electrical potential and their response to MB was interrogated via SWV. MB also has the capability to discriminate dsDNA from ssDNA, serving as a versatile redox molecule for monitoring hybridization [34]. The devices were then challenged with target DNA and the extent of hybridization was quantified via the magnitude of SWV peak signal suppression (Figure A4 and Figure A6). The percentage signal suppression in the MB reduction current due to target binding was calculated using the formula, ((*I*_probe_ − *I*_target_)/*I*_probe_) × 100, and was used for quantitative comparison of target DNA concentrations.

### 2.6. Electro-Grafting Protocol for Multiplexed Detection

A multiplexed system for detecting various targets in a sample requires immobilization of different ssDNA capture probes (different sequences) on individual electrodes of a MEA. It is not trivial, if at all possible, to selectively blot specific ssDNA capture probes on individual microelectrodes via pipetting. We accomplished this using a technique with precise control involving electric field-assisted grafting of ssDNA probes, where each electrode to be functionalized was maintained at a positive potential (with respect to the reference electrode) while the DNA capture of interests was introduced into the microfluidic channel. The positive voltage on the electrode resulted in electrophoretic migration and subsequent thiol-based immobilization of the DNA probe in the channel onto that specific electrode. The concept of electrically-guided grafting of single-stranded DNA (ssDNA) probes and multiplexed detection of target DNA in the hybrid platform is illustrated in Figure 1. Each electrode was energized individually while introducing a different DNA capture probe to create the MEA with different DNA capture probes (Figure 1A,B). Each MEA was challenged with one of the three different types of target DNA sequences (complementary to only one of the immobilized capture probes on one of the electrodes). In this sensing scheme, MB associates with the ssDNA probe and undergoes reduction. Upon hybridization, there is a decrease in the reduction peak current (signal suppression) as less MB associates with double-stranded DNA (dsDNA) [34]. Consequently, enhanced signal suppression indicates DNA hybridization, while minimal signal suppression indicates no binding. Only the electrodes having the complementary probe exhibits significant signal suppression while the other electrodes do not show any change in signal (Figure 1C,D).

We used the electro-grafting technique to create a microchannel-encapsulated np-Au MEAs (Figure A1), where micro-scale np-Au electrodes of each device were functionalized with DNA probes specific only to BRCA1, BRCA2, or p53. Multiple devices with the same electrode-probe pair configuration were fabricated to allow for three independent assessments of device performance in terms of selectivity. Each device was then challenged by introducing only one target type (BRCA1, BRCA2, or p53), and the electrode response was measured using MB reporters (see Appendix A for sequence details).

## 3. Results and Discussion

In order to study the influence of electrode nanostructure on electrically-guided grafting of ssDNA, we used np-Au as a representative nanostructured electrode, where the nanomorphology hinders the permeation of ssDNA onto the deeper surfaces. Efficient probe-grafting on such nanostructured electrodes [35] is important to reduce the necessary grafting duration, especially if individual electrodes in a MEA needs to be functionalized with different probes. We conclude with a demonstration of DNA electro-grafting on individual np-Au micro-electrodes for subsequent multiplexed detection of three different DNA targets.

### 3.1. Electrode Properties

**Figure 2:**
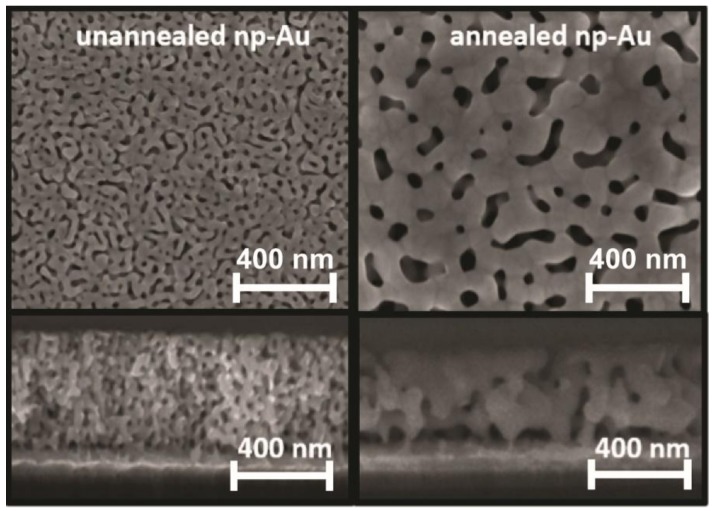
Scanning electron micrographs (SEM) of np-Au electrodes that were used for testing the influence of the electrode nanostructuring on the electro-grafting probe DNA density. Top and cross-sectional images are displayed in first and second row respectively.

Top view and cross-sectional SEM images of unannealed versus annealed np-Au electrodes in macro-scale electrochemical cell are shown in Figure 2. The film thickness was 460 ± 15 nm and 375 ± 19 nm for unannealed and annealed np-Au, respectively. Film thickness reduction after dealloying and annealing has been observed previously [36]. Median pore diameters were 24 nm and 56 nm for unannealed and annealed np-Au, respectively. In addition, the percent surface coverage by pores were 22% and 16% for unannealed and annealed np-Au, respectively. The electrochemical surface area measurements were used to calculate the surface enhancement factor (Eh), which is defined as the ratio of the effective surface area of one type of np-Au morphology to the effective surface area of the control planar Au (pl-Au) sample. These measurements confirm previous findings that the unannealed np-Au has higher surface-enhancement factor (Eh) as compared to annealed np-Au (Eh = 12, 7, 1; unannealed, annealed, planar Au respectively) [16,37].

Since the micro-scale electrodes were prepared by laser-etching of the unannealed np-Au blanket films used for macro-scale electrodes (Figure A1 and Figure A3), the film properties (i.e., thickness, pore size and coverage, surface area enhancement) remain the same. Electrochemical characterization of different electrodes of the MEA encapsulated in the channel exhibited similar electrochemical profile (CV redox peak positions and peak currents) irrespective of their proximity to the reference and counter electrodes, allowing for multiplexed biomarker detection studies to be described later.

### 3.2. Influence of Electrode Morphology on Electro-Grafting

**Figure 3:**
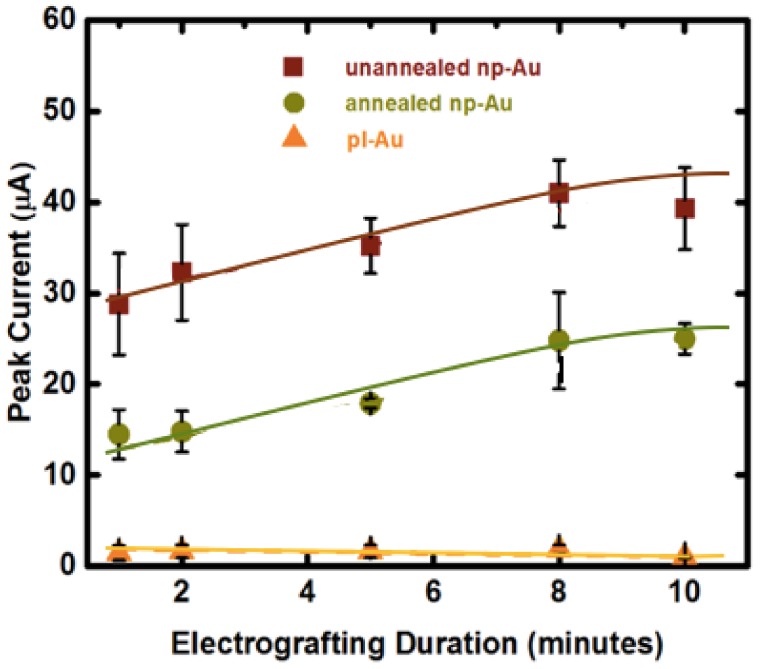
Effect of electrode morphology (unnanealed, annealed np-Au and pl-Au) and electro-grafting duration (1, 2, 5, 8, 10 min) on the peak current (surrogate for immobilized probe amount deducted from square wave voltammetry (SWV) measurements). SWV were taken at 18 Hz for unannealed np-Au, 30 Hz for annealed np-Au and 60 Hz for pl-Au after probe DNA immobilization (optimal frequency was determined previously [16]) at 10 mV/s scan rate. In contrast to the pl-Au electrodes, the np-Au electrodes require a longer electro-grafting duration to saturate the surface, as probe DNA needs to permeate the porous structure. The curve-fits are for visual guidance only.

Application of a positive voltage to individual electrodes is expected to cause swift transport and accumulation of negatively-charged DNA molecules on the working electrode [38]. While we did not observe any undesirable faradaic reactions at 0.8 V, it is plausible that the positive voltage may promote oxidative thiol-gold adsorption (reciprocal mechanism to reductive desorption used previously to detach DNA duplexes [15]), thereby enhancing the grafting process. Average SWV peak current due to MB association (used as a surrogate for immobilized probe amount) measured for the electro-grafted DNA probes was directly proportional to the available electrode surface area, suggesting a similar average probe density (number of immobilized ssDNA per unit electrochemically-accessible electrode surface area) is obtained after electro-grafting (Figure 3). This is an important result, as probe density has been shown to influence hybridization efficiency [39,40]. In addition, unlike the planar gold electrode, the average peak current increased with longer electro-grafting duration for the nanoporous electrodes, suggesting that the porous structure hinders the transport of probe DNA molecules. Interestingly, there were no differences for the electro-grafting kinetics between unannealed and annealed np-Au. Since we identified the electro-grafting process as transport-limited, one would expect annealed np-Au to reach the peak current saturation much faster than the unannealed np-Au does, due to its larger pores. However, the peak current reached its saturation during the same electro-grafting duration (10 min). We attribute this to the unannealed electrodes having smaller pores (24 nm median pore diameter) but more of them (22% surface coverage), while the annealed electrodes having larger pores (56 nm median pore diameter) yet disproportionately less of them (16% surface coverage). In this explanation, the larger pores provide less-hindered transport of target DNA and larger percent surface coverage provides more influx area. Finally, perhaps the point that is of greatest significance to the preparation of probe DNA-immobilized nanostructured multiple electrode arrays is that passive probe immobilization (under no applied electric field) on np-Au requires at least a 2 h-long incubation (typically an overnight incubation of 15 h is used), which is shortened by 25-fold to 10 min by using electro-grafting.

### 3.3. Hybridization Efficiency for Microelectrodes

**Figure 4:**
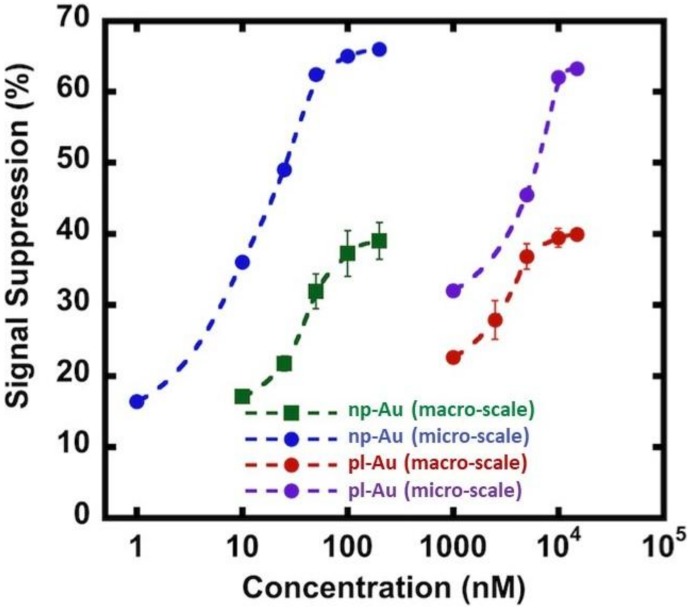
Comparison of DNA hybridization efficiency between macro-scale and micro-scale electrode configurations for np-Au and pl-Au electrodes. Signal suppression (%) is defined as ((*I*_probe_ − *I*_target_)/*I*_probe_) × 100, where *I*_probe_ and *I*_target_ are SWV current signals measured for probe DNA alone and upon target capture, respectively. Raw data are included in Figure A5 and Figure A6.

As the next step in multiplexing the detection of nucleic acid biomarkers, we fabricated an electrode array and encapsulated them in microfluidic channels to reduce analyte use and facilitate the interrogation of multiple electrodes. In order to accommodate multiple distinct electrodes, we reduced the electrode diameter to 0.3 mm from 4 mm-diameter macro-scale electrodes, which translates to a geometric area reduction of ~170 fold. We used the unannealed np-Au electrode morphology since there was no significant difference in the grafting kinetics between the annealed and unannealed morphologies. We should note that the electrode morphologies can be individually modified to attain optimal detection ranges for different sensing conditions [18]. Specifically, the np-Au macro-scale electrochemical devices displayed a dynamic range of detection between 10–200 nM, as observed in previous studies [16,17]. The detection range shifted to 1 nM and the signal suppression increased by nearly two times in the case of the MEAs encapsulated in the microfluidic channel (Figure 4). The enhanced sensor performance of the microelectrodes encapsulated in microchannels is due to multiple factors. In the case of macro-scale electrodes, the transport perpendicular to the electrode is in the form of a semi-infinite planar diffusion. In contrast, for micro-scale electrodes, a radial diffusion field is quickly attained, which results in a higher current density [33]. In addition to this diffusion field-based enhancement (i.e., enhanced mass transport for the case of micro-scale electrode), the background current that is associated with the electric double layer charging (capacitive current) scales with the electrode surface area, where the reduced electrode footprint lowers the background current [41,42]. The combination of enhanced mass transport and reduced capacitive currents in miniaturized electrodes translates into increased signal-to-noise ratio allowing for more sensitive analysis. Similar enhancement in performance was observed for pl-Au counterparts (Figure 4) when used in micro- versus macro-scale electrode configurations (Figure 4). Taken together, the electrically-guided electrode functionalization method allowed for creating a MEA with each of its electrodes displaying a different DNA capture probe with minimal cross-contamination by other capture probes.

### 3.4. Multiplexed Detection of Cancer Markers

**Figure 5:**
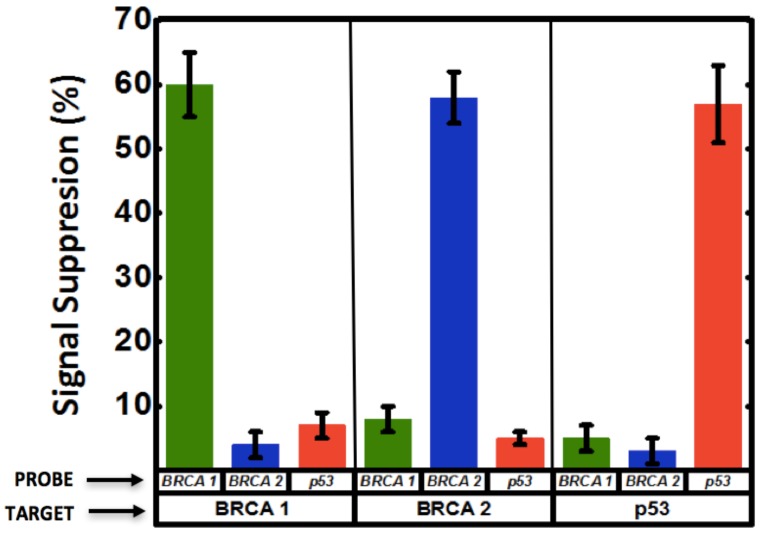
Multiplexed detection of breast cancer-related biomarkers. Three different DNA capture probes (BRCA1, BRCA2 and p53) were grafted on three adjacent electrodes encapsulated in a microfluidic channel. SWV of the interaction of grafted probe DNA with redox marker methylene blue (MB) is measured, as well as the SWV response of each of the electrodes to the different target sequences introduced (BRCA1, BRCA2 and p53) to each microfluidic device. Significant signal suppression was observed when the sample contained the target sequence corresponding to the electrode being interrogated via SWV, while the other two electrodes showed minimal change in signal. The signal suppression observed for all electrodes in response to the targets is summarized as a bar graph.

**Figure A7:**
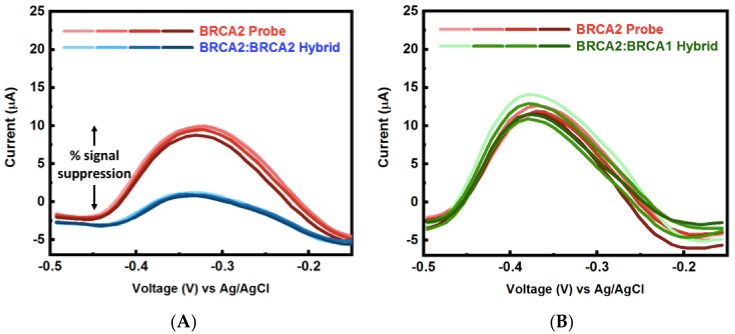
Example SWV raw data for BRCA2 probe and (**A**) BRCA2:BRCA2 hybrid perfect-match, (**B**) BRCA2:BRCA1 mismatch hybrid current signal in the microfluidic channel used to generate Figure 5—Multiplexed detection of breast cancer-related biomarkers.

Circulating cell-free nucleic acids with concentrations ranging between 0 to 2 µg/mL have shown promise as biomarkers for early cancer diagnosis and surveillance [43]. This concentration range corresponds to approximately 0 to 300 nM for 20-mer ssDNA (similar molecular size used in this study), which falls well within the range of detection of the np-Au sensor demonstrated here. In addition, identification of multiple biomarkers drastically improves accuracy in disease diagnosis [3,26]. To that end, motivated by the advantages of np-Au as a working electrode for the probe DNA electro-grafting and microfluidics as the sensing platform, the two were combined to create the multiplexed system for DNA detection. Multiplexed electrochemical detection of breast cancer biomarkers BRCA1, BRCA2, p53, was achieved as a result of the selective functionalization and hybridization as schematically introduced in Figure 1. A significant signal suppression (~60%) was observed for the electrode with successful hybridization. The other two electrodes having mismatched probe sequences showed minimal change in signal, indicating no binding (Figure 5 and Figure A7), suggesting both high selectivity and high location-specificity in immobilization of the probes (i.e., minimal cross-contamination during the electro-grafting step). For example, BRCA1 probe was grafted on electrode 1. When the sample contained BRCA1 target, this electrode resulted in increased signal suppression indicating hybridization, while minimal change in signal was observed on this electrode when the sample contained BRCA2 and p53 targets. The other two electrodes in the channel functionalized with BRCA2 and p53 were also interrogated via SWV after BRCA1 target binding and MB incubation. These electrodes displayed minimal signal suppression in response to BRCA1 target. Similarly, samples containing BRCA2 and p53 targets were introduced to different devices containing each of the three probes on three different electrodes and the SWV response was measured. The signal suppression obtained from these measurements is summarized in Figure 5. Essentially, enhanced signal suppression was only observed for electrodes with a matched probe-target pair, while the response from the other two electrodes remained mostly unchanged, suggesting very high selectivity in both electrode functionalization and detection steps, as well as reproducibility between different devices.

## 4. Conclusions

We demonstrated that the immobilization kinetics depend strongly on the electrode morphology (planar versus nanostructured). The electro-grafting reduced probe immobilization duration by 25-fold for np-Au electrodes in comparison to conventional immobilization under no electric field. In addition, the electrically-guided DNA grafting technique allowed for detection of multiple DNA targets with different sequences when combined with the nanostructured electrodes in a microfluidic setup. The resulting microfluidic platform yielded enhanced signal suppression and an order of magnitude improvement in detection limit compared to the traditional macro-scale electrochemical cell. This was mainly attributed to the rapid achievement of a radial diffusion field that improves mass transport to the micro-scale electrode and reduces capacitive currents, resulting in the increased signal-to-noise ratio allowing for more sensitive analysis. Distinct DNA probes were electrically grafted on individual electrodes of the array by applying a positive potential to the electrode of interest and the MEA generated was employed to achieve multiplexed detection of three cancer biomarkers (at physiologically relevant concentrations [43]) with high selectivity and sensitivity. We expect these findings to assist in developing multiplexed detection platforms for a diverse set of applications, including multiplexed aptamers for protein or small-molecule sensing.

## Figures and Tables

**Figure A5:**
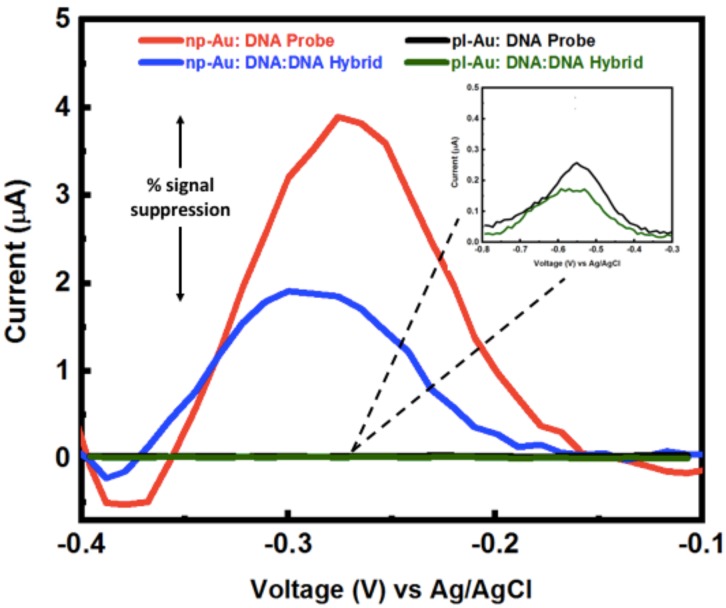
Example SWV raw data for DNA probe and DNA:DNA hybrid current signal in the microfluidic channel for unnanealed np-Au and pl-Au electrodes used to generate Figure 4—Comparison of DNA hybridization efficiency between macro-scale and micro-scale cell sensor configurations for np-Au and pl-Au electrodes.

**Figure A8:**
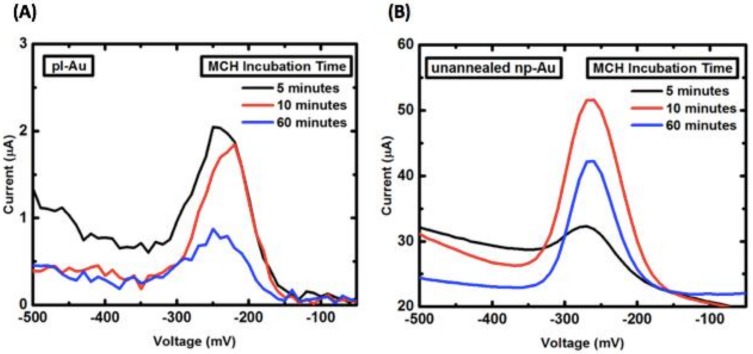
Influence of mercaptohexanol (MCH) incubation duration on the probe current for (**A**) pl-Au and (**B**) np-Au electrodes. For both morphologies optimal MCH incubation duration ranges between 10 to 60 min.

**Figure A9:**
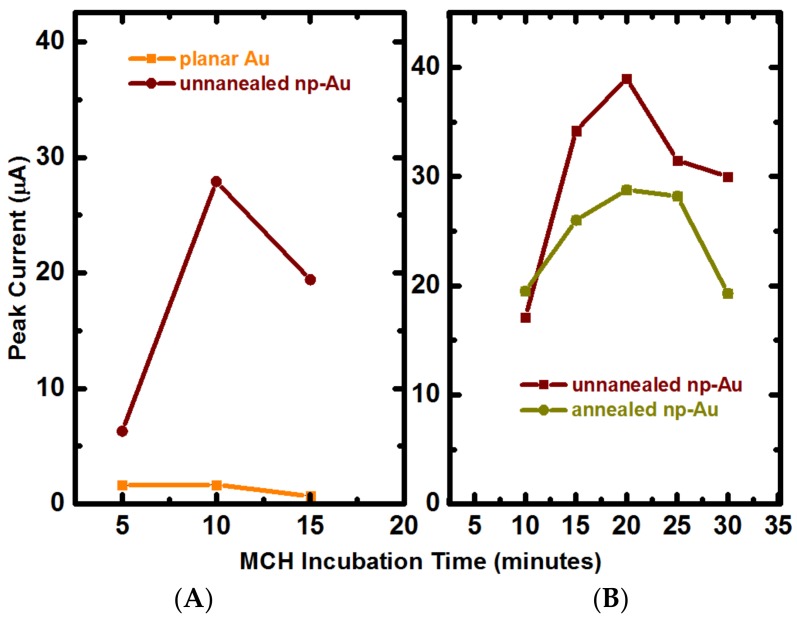
Influence of MCH incubation duration on the peak probe DNA current for different morphologies, where the incubation duration preceding the reduction in peak current indicates the optimal duration for comparing the amount of immobilized probe DNA on different electrode morphologies. (**A**) 10 min was identified as the optimal MCH incubation duration for comparing pl-Au and unannealed np-Au. (**B**) 20 min was the optimal MCH incubation duration for comparing unannealed np-Au and annealed np-Au.

## Appendix A

### Appendix A.1. Overview of the Integrated Device and Macro-Scale Electrochemical Cell

Figure A1 is an optical image of the integrated microfluidic device, which is composed of two layers. The bottom layer is a 1 mm-thick glass slide with nanoporous gold (np-Au) microelectrode patterns and the top layer is composed of polydimethylsiloxane (PDMS) microfluidic channels. The microfluidic layer is bonded to the glass slide (encapsulating the microelectrodes) via oxygen plasma activation of the surfaces. There are two separate microfluidic channels on a single chip with 125 µm-high and 2 mm-wide channels. The shape of the microfluidic channel is designed to reduce dead volume in the corners. Four np-Au working micro-scale electrodes with 300 µm-diameter are embedded on the base of each microfluidic channel. Each individual microelectrode is electrically addressable through a 3 mm × 3 mm peripheral contact pad, where the microelectrode and contact pad are connected via 60 µm-wide traces.

### Appendix A.2. Fabrication of Nanoporous Gold Electrodes

The representative schematics of a macro-scale and microfluidic np-Au electrodes within electrochemical cell are shown in Figure A2.

### Appendix A.3. Fabrication of Microfluidic Channels

For the PDMS microfluidic channel fabrication, a novel rapid prototyping method was used (Figure A3). Instead of using conventional SU8 photolithography to prepare the master substrate for soft lithography, a 132 µm-thick self-adhesive aluminum foil was used for a rapid prototyping. The foil was laminated onto the substrate (plastic petri dish) and cut into the designed shape under 355 nm ultraviolet (UV) laser ablation. The foil was then peeled off leaving behind the patterned channel. The premixed PDMS at 1:10 ratio (crosslinking agent: base elastomer, by weight) was poured onto the mold in a petri dish and cured at 85 °C for three hours on a hot plate. Following the curing step, a 0.15 mm-diameter biopsy punch was used to make the inlet and outlets holes at the ends of each PDMS microchannel. The glass slides with np-Au microelectrodes was coupled with the PDMS microfluidic channels via oxygen plasma treatment and alignment marks. The plasma treatment was performed in a plasma chamber (Techniques REI) at 30 W (5%) power under 0.5 Torr chamber pressure for 12 s with an oxygen flow rate of 20 sccm. After bonding, the device was kept at 70 °C on a hot plate for 10 min to promote a stronger bond between glass and PDMS.

### Appendix A.4. Preparation of DNA Sensor

The DNA sequences used in this study were purchased from Integrated DNA Technologies, USA. Incubation in a 5 mM TCEP solution for 2 h was used to reduce the thiolated probe DNA stock and excess TCEP was filtered out. TCEP provided a higher yield of reduced DNA and a simpler filtration protocol in comparison to reagents such as dithiothreitol. The 5-prime end of the single-stranded DNA (ssDNA) probe was modified with a C6 linker and a thiol group. The sequences were:

Macro-scale vs. microfluidic-encapsulation comparison:

Probe ssDNA: 5ThioMC6-D/CGT GTT ATA AAA TGT AAT TTG GAA TT;
Target DNA: AAT TCC AAA TTA CAT TTT ATA ACA CG

Multiplexed detection:

BRCA1 probe: 5ThioMC6-D/GATTTTCTTCCTTTTGTTC
BRCA2 probe: 5ThioMC6-D/TACGGCCCTGAAGTACA
p53 probe: 5ThioMC6-D/TCCTCCGGTTCATGCCA
BRCA1 target: GAACAAAAGGAAGAAAATC
BRCA2 target: TGTACTTCAGGGCCGTA
p53 target: TGGCATGAACCGGAGGA

*Passive (no electric field) probe-grafting*: The np-Au and planar gold macro-scale electrodes were cleaned in dilute (1:4) piranha solution for 20 s and plasma treated for 1 min prior to functionalization. The microchannel-encapsulated MEAs were degassed for 10 min in a vacuum desiccator and plasma cleaned for 5 min prior to functionalization. The devices were then incubated in 25 mM phosphate buffer (PB), containing 2 µM thiolated probe DNA and 50 mM MgCl_2_ for 15 h at room temperature. 1 mM mercaptohexanol (MCH) prepared in PB was used as back-fill agent to passivate the surface that were not covered by the probe DNA. The electrodes were thoroughly rinsed with PB to remove non-specifically bound DNA.

*Electro-grafting*: The microfluidic channel was filled with 25 mM phosphate buffer (PB), containing 2 µM thiolated probe DNA and 50 mM MgCl_2_. Open circuit potential was measured to ensure connectivity. 0.8 V was applied to the electrode to-be-functionalized with respect to a Ag/AgCl reference electrode for 10 min to electrophoretically attract DNA probe onto the electrode. The channel was then rinsed with PB to remove non-specifically bound probe molecules. This potential did not produce undesirable faradaic reactions and provided fast electrophoretic movement onto the electrodes.

DNA-functionalized devices (both macro-scale and micro-scale electrodes) were incubated with 20 µM methylene blue (MB) prepared in 1X phosphate buffered saline (PBS) 10 min. Unbound MB molecules were removed by washing with PB. For the macro-scale experiments, the electrode was placed inside a custom-built Teflon electrochemical cell and 1X PBS was used as the electrolyte for measurements. In the case of the integrated devices, the channel was filled with 1X PBS for measurements. Probe-modified devices were interrogated with the target DNA of interest. The devices were incubated with desired target DNA prepared in PB containing 50 mM MgCl_2_ for 35 min at 37 °C. Non-specifically-bound target molecules were removed by a PB rinse.

## Appendix B

### Appendix B.1. Electrochemical Methods and Square Wave Voltammetry (SWV) Raw Data

All the electrochemical measurements were carried out using a Gamry Reference 600 potentiostat. The np-Au and pl-Au electrodes with footprints of 0.15 cm^2^ were used for macro-scale experiments. A homemade Teflon cell was utilized to carry out electrochemical measurements in macro-scale while platinum wire and Ag/AgCl electrodes were used as counter and reference electrodes, respectively. In case of the integrated devices, a flow through Ag/AgCl electrode placed in the inlet of the microfluidic channel served as the reference electrode while platinum wire counter electrode was inserted into the outlet of the microfluidic channel. Probe-grafting and target hybridization were electrochemically quantified using the MB-DNA reduction peak obtained via square wave voltammetry (SWV). All SWV measurements were performed in 1X PBS over the potential range 0 to −0.5 V with an amplitude of 40 mV. Step size of 4 mV and frequency of 18 Hz for unnanealed np-Au, 30 Hz for annealed np-Au and 60 Hz for pl-Au were used respectively for macro-scale devices. The ionic transport to electrodes with different morphologies were responsible for the distinct optimal frequencies. For a discussion of the underlying reasons for different optimal SWV frequencies, please refer to our previous work [16]. The step size was increased to 10 mV for the microchannel-encapsulated MEAs to reduce the noise. 30 Hz and 60 Hz frequencies were used for np-Au and pl-Au respectively for the microchannel-encapsulated MEAs.

Figure A4 illustrates the influence of electro-grafting duration at two extreme time points (i.e., 2 and 10 min) on probe-grafting (indicative of surface coverage of ssDNA capture probe) for three different electrode morphologies (i.e., planar gold, np-Au with coarser pores yet less effective surface area, np-Au with finer pores yet larger effective surface area).

Next, Figure A5 demonstrates peak current before and after hybridization in the microfluidic channel for planar gold and nanoporous gold electrodes. The inset shows the details of the pl-Au peak currents, which are significantly smaller than that for np-Au leading to a less sensitive device.

Figure A6 shows that the MAEs were successfully functionalized with the DNA probe, as indicated by the reduction peak of MB at around −275 mV. More importantly, to determine the detection limit of the MEAs, the probe-functionalized nanostructured sensors were challenged with different concentrations of target DNA, where a decrease in the MB signal indicates successful hybridization. MAEs showed 10-fold increase in the limits of detection (1 nM target concentration was successfully detected) as compared to the macro-scale np-Au electrodes [16].

Finally, Figure A7 contrasts the peak currents before and after introducing the ssDNA targets. Figure A7A demonstrates hybridization to perfect sequence-match (hence signal suppression) and the Figure A7B demonstrates the complete mismatch (hence no signal suppression). In addition, the n = 4 for each case shows high reproducibility, where each trial is a different electrode.

### Appendix B.2. Mercaptohexanol (MCH) Incubation Optimization for Different np-Au Morphologies

Typically, ssDNA probes are attached to the gold surface through a sulfur-gold linkage and they form a self-organized layer. DNA nucleotides can presumably adsorb to gold via multiple amine moieties, as amines are known to chemisorb weakly to gold surfaces. Such adsorption at multiple sites can interfere with hybridization of the immobilized strands [44]. A small-molecule blocking agent, 6-mercapto-1-hexanol (MCH), has been commonly used to treat surfaces to enable better accessibility of immobilized probes to complementary target sequences. The thiol group of MCH rapidly displaces the weaker adsorptive contacts between DNA nucleotides and the substrate, leaving the probes tethered primarily through their thiol end-groups [44]. After MCH treatment, the initially compact ssDNA swells and extends further into the solution. The less-constrained tethering conformation renders the probes highly accessible to target DNA and improves hybridization efficiency. Even though the interactions of MCH with immobilized nucleic acids have been well studied and the system has been optimized under many different conditions [16], MCH incubation time for the electro-grafted probe DNA has to be optimized.

A commonly used duration for the sample incubation with MCH after passive probe DNA immobilization is 2 h [16]. However, under electro-grafting conditions, these durations were too long, resulting in probe DNA displacement as judged by reduction in probe current (Figure A8). Long MCH incubation durations were particularly detrimental to probe DNA immobilized on pl-Au electrodes, plausibly due to unhindered access of MCH to the surface in contrast to the hindered transport for np-Au electrodes (Figure A8A).

In order to properly compare pl-Au probe DNA grafting density to the other morphologies (e.g., np-Au), a series of 1 min electro-grafted samples were tested under different MCH incubation duration in order to identify the optimal duration, which is right before the peak current begins to decrease. Identification of this duration should enable proper estimation of probe DNA grafting amount. To that end, a 10-min MCH incubation was used for all subsequent experiments comparing pl-Au and unannealed np-Au (Figure A9A).

The MCH incubation time had to be further optimized for the comparison between unannealed np-Au and annealed np-Au electrodes. It was observed that 10 min-long incubation was not enough to entirely back-fill the electrode surfaces, which would have led to erroneous determination of the amount of probe DNA immobilized. A 20 min-long incubation was identified as optimal and was used for comparing the probe DNA immobilization on these electrode morphologies (Figure A9B).

## References

[1] Cederquist K.B., Kelley S.O. (2012). Nanostructured biomolecular detectors: Pushing performance at the nanoscale. Curr. Opin. Chem. Biol..

[2] Wang L., Li P.C.H. (2011). Microfluidic DNA microarray analysis: A review. Anal. Chim. Acta.

[3] Goldhirsch A., Wood W.C., Coates A.S., Gelber R.D., Thurlimann B., Senn H.J., Panel M. (2011). Strategies for subtypes-dealing with the diversity of breast cancer: Highlights of the St Gallen International Expert Consensus on the Primary Therapy of Early Breast Cancer 2011. Ann. Oncol..

[4] Sassolas A., Leca-Bouvier B.D., Blum L.J. (2008). DNA biosensors and microarrays. Chem. Rev..

[5] Cheek B.J., Steel A.B., Torres M.P., Yu Y.Y., Yang H.J. (2001). Chemiluminescence detection for hybridization assays on the flow-thru chip, a three-dimensional microchannel biochip. Anal. Chem..

[6] Lord H., Kelley S.O. (2009). Nanomaterials for ultrasensitive electrochemical nucleic acids biosensing. J. Mater. Chem..

[7] Daniel S., Rao T.P., Rao K.S., Rani S.U., Naidu G.R.K., Lee H.Y., Kawai T. (2007). A review of DNA functionalized/grafted carbon nanotubes and their characterization. Sens. Actuators B Chem..

[8] Gasparac R., Taft B.J., Lapierre-Devlin M.A., Lazareck A.D., Xu J.M., Kelley S.O. (2004). Ultrasensitive electrocatalytic DNA detection at two- and three-dimensional nanoelectrodes. J. Am. Chem. Soc..

[9] Bin X.M., Sargent E.H., Kelley S.O. (2010). Nanostructuring of Sensors Determines the Efficiency of Biomolecular Capture. Anal. Chem..

[10] Soleymani L., Fang Z.C., Sargent E.H., Kelley S.O. (2009). Programming the detection limits of biosensors through controlled nanostructuring. Nat. Nanotechnol..

[11] Park S., Kim H.C., Chung T.D. (2012). Electrochemical analysis based on nanoporous structures. Analyst.

[12] Joo S., Park S., Chung T.D., Kim H.C. (2007). Integration of a nanoporous platinum thin film into a microfluidic system for non-enzymatic electrochemical glucose sensing. Anal. Sci..

[13] Das J., Ivanov I., Montermini L., Rak J., Sargent E.H., Kelley S.O. (2015). An electrochemical clamp assay for direct, rapid analysis of circulating nucleic acids in serum. Nat. Chem..

[14] Das J., Kelley S.O. (2013). Tuning the Bacterial Detection Sensitivity of Nanostructured Microelectrodes. Anal. Chem..

[15] Daggumati P., Appelt S., Matharu Z., Marco M.L., Seker E. (2016). Sequence-Specific Electrical Purification of Nucleic Acids with Nanoporous Gold Electrodes. J. Am. Chem. Soc..

[16] Daggumati P., Matharu Z., Seker E. (2015). Effect of Nanoporous Gold Thin Film Morphology on Electrochemical DNA Sensing. Anal. Chem..

[17] Daggumati P., Matharu Z., Wang L., Seker E. (2015). Biofouling-Resilient Nanoporous Gold Electrodes for DNA Sensing. Anal. Chem..

[18] Matharu Z., Daggumati P., Wang L., Dorofeeva T.S., Li Z.D., Seker E. (2017). Nanoporous-Gold-Based Electrode Morphology Libraries for Investigating Structure-Property Relationships in Nucleic Acid Based Electrochemical Biosensors. ACS Appl. Mater. Interfaces.

[19] Ramanaviciene A., Ramanavicius A. (2004). Pulsed amperometric detection of DNA with an ssDNA/polypyrrole-modified electrode. Anal. Bioanal. Chem..

[20] Hong J., Edel J.B., de Mello A.J. (2009). Micro- and nanofluidic systems for high-throughput biological screening. Drug Discov. Today.

[21] Edman C.F., Raymond D.E., Wu D.J., Tu E.G., Sosnowski R.G., Butler W.F., Nerenberg M., Heller M.J. (1997). Electric field directed nucleic acid hybridization on microchips. Nucl. Acids Res..

[22] Fixe F., Branz H.M., Louro N., Chu V., Prazeres D.M.F., Conde J.P. (2005). Electric-field assisted immobilization and hybridization of DNA oligomers on thin-film microchips. Nanotechnology.

[23] Gultepe E., Nagesha D., Sridhar S., Amiji M. (2010). Nanoporous inorganic membranes or coatings for sustained drug delivery in implantable devices. Adv. Drug Deliv. Rev..

[24] Howorka S., Siwy Z. (2009). Nanopore analytics: Sensing of single molecules. Chem. Soc. Rev..

[25] Huber P. (2015). Soft matter in hard confinement: phase transition thermodynamics, structure, texture, diffusion and flow in nanoporous media. J. Phys. Condens. Matter.

[26] Finch A., Metcalfe K., Lui J., Springate C., Demsky R., Armel S., Rosen B., Murphy J., Elit L., Sun P. (2009). Breast and ovarian cancer risk perception after prophylactic salpingo-oophorectomy due to an inherited mutation in the BRCA1 or BRCA2 gene. Clin. Genet..

[27] Levine A.J. (1997). p53, the cellular gatekeeper for growth and division. Cell.

[28] Li Z., Seker E. (2017). Configurable microfluidic platform for investigating therapeutic delivery from biomedical device coatings. Lab Chip.

[29] Erlebacher J., Aziz M.J., Karma A., Dimitrov N., Sieradzki K. (2001). Evolution of nanoporosity in dealloying. Nature.

[30] Li Z.D., Hu D., Zhao Z.T., Zhou M.Y., Liu R., Lo J.F. (2015). Balancing oxygen diffusion and convection in spiral microfluidics to mimic radial biological gradients. Biomed. Microdevices.

[31] Matharu Z., Bandodkar A.J., Gupta V., Malhotra B.D. (2012). Fundamentals and application of ordered molecular assemblies to affinity biosensing. Chem. Soc. Rev..

[32] Scanlon M.D., Salaj-Kosla U., Belochapkine S., MacAodha D., Leech D., Ding Y., Magner E. (2011). Characterization of nanoporous gold electrodes for bioelectrochemical applications. Langmuir.

[33] Heinze J. (1993). Ultramicroelectrodes in electrochemistry. Angew. Chem. Int. Ed..

[34] Kerman K., Ozkan D., Kara P., Meric B., Gooding J.J., Ozsoz M. (2002). Voltammetric determination of DNA hybridization using methylene blue and self-assembled alkanethiol monolayer on gold electrodes. Anal. Chim. Acta.

[35] Collinson M.M. (2013). Nanoporous gold electrodes and their applications in analytical chemistry. ISRN Anal. Chem..

[36] Seker E., Gaskins J., Bart-Smith H., Zhu J., Reed M., Zangari G., Kelly R., Begley M. (2007). The effects of annealing prior to dealloying on the mechanical properties of nanoporous gold microbeams. Acta Mater..

[37] Kurtulus O., Daggumati P., Seker E. (2014). Molecular release from patterned nanoporous gold thin films. Nanoscale.

[38] Wong I.Y., Melosh N.A. (2009). Directed hybridization and melting of DNA linkers using counterion-screened electric fields. Nano Lett..

[39] Peterson A.W., Heaton R.J., Georgiadis R.M. (2001). The effect of surface probe density on DNA hybridization. Nucl. Acids Res..

[40] Ricci F., Lai R.Y., Heeger A.J., Plaxco K.W., Sumner J.J. (2007). Effect of molecular crowding on the response of an electrochemical DNA sensor. Langmuir.

[41] Soleymani L., Li F. (2017). Mechanistic challenges and advantages of biosensor miniaturization into the nanoscale. ACS Sens..

[42] Schmueser I., Walton A.J., Terry J.G., Woodvine H.L., Freeman N.J., Mount A.R. (2013). A systematic study of the influence of nanoelectrode dimensions on electrode performance and the implications for electroanalysis and sensing. Faraday Discuss..

[43] Schwarzenbach H. (2013). Circulating nucleic acids as biomarkers in breast cancer. Breast Cancer Res..

[44] Steel A.B., Levicky R.L., Herne T.M., Tarlov M.J. (2000). Immobilization of nucleic acids at solid surfaces: Effect of oligonucleotide length on layer assembly. Biophys. J..

